# Amantadine Toxicity in *Apostichopus japonicus* Revealed by Proteomics

**DOI:** 10.3390/toxics11030226

**Published:** 2023-02-27

**Authors:** Junqiang Zhao, Jianqiang Chen, Xiuhui Tian, Lisheng Jiang, Qingkui Cui, Yanqing Sun, Ningning Wu, Ge Liu, Yuzhu Ding, Jing Wang, Yongchun Liu, Dianfeng Han, Yingjiang Xu

**Affiliations:** 1 Yantai Key Laboratory of Quality and Safety Control and Deep Processing of Marine Food, Shandong Key Laboratory of Marine Ecological Restoration, Shandong Marine Resource and Environment Research Institute, Yantai 264006, China; 2 School of Food, Shanghai Ocean University, Shanghai 200120, China; 3 Qingdao Ocean Management Security Center, Qingdao 266000, China; 4 Laizhou Marine Development and Fisheries Service Center, Yantai 261499, China

**Keywords:** *Apostichopus japonicus*, amantadine, proteomics, histological damage, enzymatic reactions

## Abstract

Amantadine exposure can alter biological processes in sea cucumbers, which are an economically important seafood in China. In this study, amantadine toxicity in *Apostichopus japonicus* was analyzed by oxidative stress and histopathological methods. Quantitative tandem mass tag labeling was used to examine changes in protein contents and metabolic pathways in *A. japonicus* intestinal tissues after exposure to 100 µg/L amantadine for 96 h. Catalase activity significantly increased from days 1 to 3 of exposure, but it decreased on day 4. Superoxide dismutase and glutathione activities were inhibited throughout the exposure period. Malondialdehyde contents increased on days 1 and 4 but decreased on days 2 and 3. Proteomics analysis revealed 111 differentially expressed proteins in the intestines of *A. japonicus* after amantadine exposure compared with the control group. An analysis of the involved metabolic pathways showed that the glycolytic and glycogenic pathways may have increased energy production and conversion in *A. japonicus* after amantadine exposure. The NF-κB, TNF, and IL-17 pathways were likely induced by amantadine exposure, thereby activating NF-κB and triggering intestinal inflammation and apoptosis. Amino acid metabolism analysis showed that the leucine and isoleucine degradation pathways and the phenylalanine metabolic pathway inhibited protein synthesis and growth in *A. japonicus.* This study investigated the regulatory response mechanisms in *A. japonicus* intestinal tissues after exposure to amantadine, providing a theoretical basis for further research on amantadine toxicity.

## 1. Introduction

Sea cucumbers belong to the invertebrate class Echinodermata and are common marine invertebrates [1]. The sea cucumber *Apostichopus japonicus* is one of the most commonly found and economically valuable species in China. It is rich in amino acids, fatty acids, collagen, saponins, and other bioactive components, giving it high nutritional and medicinal value. In recent years, the industrial farming of sea cucumber in China has rapidly developed owing to market demand. The quality of farmed sea cucumber is highly influenced by the quality of the seawater in which it is grown, and the product can be compromised if the seawater is contaminated by environmental pollutants, including human medications [2].

Amantadine is a drug used to treat movement disorders related to Parkinson’s disease. It has also been used as an influenza antiviral and has been used to control influenza virus in livestock farming because of its low cost and its ability to effectively inhibit influenza virus [3,4]. However, the illegal overuse of amantadine in the livestock industry has exacerbated its accumulation in the environment, thus harming water ecology [5,6]. Some marine organisms have been shown to be contaminated with amantadine [7,8]. The Ministry of Agriculture of China issued Circular 560, which banned the use of human antivirals for veterinary purposes. However, amantadine has still been detected in seawater and in *A. japonicus* at concentrations of up to 140 ng/L and 4.3 µg/kg, respectively [5,6,9].

Cellular apoptosis and the functional development of organisms can be affected by amantadine [10,11]. At high amantadine concentrations in the human body, there is a risk of poisoning and tissue damage [12,13,14]. However, little is known about the effects of amantadine in aquatic products or the toxic effects of amantadine in *A. japonicus* [15,16]. Therefore, the toxic-response mechanisms and biological effects of amantadine in *A. japonicus* should be characterized. Proteomics is a core technology in current post-genomic systems biology approaches and is used to understand molecular mechanisms and identify key diagnostic and prognostic biomarkers. Proteomics has rapidly developed in recent years. Tandem mass tag (TMT)-based quantitative proteomics is one of the most powerful methods for the quantitative analysis of differentially expressed proteins; this method has high throughput and low systematic error [17].

In this study, the effect of amantadine on the intestinal structure of *A. japonicus* was investigated at an exposure concentration of 100 µg/L. The antioxidant capacities of intestinal tissues were investigated using superoxide dismutase (SOD), catalase (CAT), glutathione (GSH), and malondialdehyde (MDA) as reference indicators. In addition, TMT quantitative proteomics was used to analyze proteomic differences and to screen for relevant key proteins and metabolic pathways to examine the response mechanisms. The findings provide a basis for understanding the response mechanisms of *A. japonicus* to amantadine exposure.

## 2. Materials and Methods

### 2.1. Experimental Animals

Artificially bred *A. japonicus* were purchased from the Shandong Anyuan Seed Technology Co., Ltd. (Yantai, China), and the organisms had an average weight of 50 ± 3.0 g. Healthy, vigorous *A. japonicus* were selected and maintained in the laboratory for 7 days in a 60 L rectangular glass tank filled with continuously oxygenated water at 13–15 °C. Half of the water was changed daily. After the initial 7-day maintenance period, the *A. japonicus* were cleaned of fecal matter and fasted during a transition period; they were also fasted during the subsequent exposure experiment. At the end of the transition period, healthy and vigorous *A. japonicus* were randomly selected for the exposure experiment. Fresh, clean seawater was used for the experiment. Amantadine (≥98% pure) was purchased from the Shanghai Maclean Biochemical Technology Co., Ltd. (Shanghai, China), and a 100 mg/L stock solution was prepared and stored at 4 °C. Two groups were used for the experiment, a control group maintained in normal seawater and an experimental group exposed to amantadine. There were four parallel groups each for the control group and the experimental group. Each tank was filled with 30 L of seawater. The amantadine stock solution was added to a final concentration of 100 µg/L in the experimental group tank. Each group contained approximately 15 individual *A. japonicus*. The exposure duration was 96 h.

### 2.2. Sample Preparation

At the end of the exposure experiment, six *A. japonicus* from four parallel groups were randomly selected and dissected on ice. Each gut was equally divided into 2 mL lyophilization tubes. Three parallel samples from both the control and experimental groups were used to analyze enzyme activities, and four parallel samples from each group were used for proteomics. After collection, the samples were snap-frozen in liquid nitrogen and stored at −80 °C until analysis.

### 2.3. Histological and Enzymatic Activity Analyses

On days 0 and 4, tissue samples from the intestinal tracts of *A. japonicus* were fixed for 24 h in a 10-fold volume of Bouin’s solution and then transferred to 70% alcohol for storage. The fixed tissues were dehydrated in an ethanol gradient (70%, 80%, 95%, 100%), made transparent in xylene, submerged in wax, and embedded in paraffin using an embedding machine. A microtome was used to trim and cut 6 µm slices, which were then baked at 60 °C after patching, stained with an H-E automatic staining machine, and sealed with neutral gum. The samples were observed under a light microscope and photographed for documentation. To determine the enzyme activity, 0.20 g of tissue was mixed with 0.75% cold saline at a 1:9 weight-to-volume ratio and homogenized in an ice bath using a portable high-speed disperser. The homogenized tissue was centrifuged for 10 min (4 °C, 2000 r/min), and the supernatant was collected to analyze the SOD and CAT enzyme activities and the MDA and GSH contents using a kit purchased from the Nanjing Jiancheng Institute of Biological Engineering.

### 2.4. TMT-Based Quantitative Proteomics Analysis

#### 2.4.1. Total Protein Extraction

The tissue samples were removed from −80 °C storage, ground to a powder at low temperature, quickly transferred to a centrifuge tube that was precooled with liquid nitrogen, and 600 µL of lysis buffer (50 mM Tris buffer, 8 M urea, 1% SDS, pH = 8) was added and mixed with shaking. The samples were then sonicated for 5 min to achieve full lysis (2 s on/3 off) and centrifuged for 20 min (13,000 r/min, 4 °C). The supernatant was collected and precipitated by adding four times the volume of cold acetone (containing 10 mM DTT) and incubated for 2 h. The precipitate was collected by centrifugation (20 min, 13,000 r/min, 4 °C). To wash the sample, the precipitate was resuspended in 800 µL of cold acetone (containing 10 mM DTT) and centrifuged (20 min, 13,000 r/min, 4 °C). The precipitate was then air-dried. The total protein was quantified using the Bradford assay. A 20 µg aliquot of protein was analyzed by SDS-PAGE to evaluate the quality of the total protein.

#### 2.4.2. TMT Labeling of Peptides

DB dissolution buffer (8 M urea (Sinopharm, Shanghai, China), 100 mM TEAB (Sigma, Hanover, Germany), pH 8.5) was added to each protein sample to a final volume of 100 µL. Next, 100 mM TEAB buffer (Sigma, Hanover, Germany) and trypsin (Promega, Madison, WI, USA) were added, and the samples were mixed and digested at 37 °C for 4 h. After adding additional trypsin and CaCl_2_, the samples were further digested overnight. Formic acid was then added to bring the pH to <3, and the samples were centrifuged at 12,000× *g* for 5 min at room temperature. The supernatant was gradually fed into a C18 desalination column and washed three times with washing buffer (0.1% formic acid (Thermo Fisher Scientific, Bremen, Germany), 3% acetonitrile (Thermo Fisher Scientific, Bremen, Germany)) and then eluted with elution buffer (0.1% formic acid (Thermo Fisher Scientific, Bremen, Germany), 70% acetonitrile (Thermo Fisher Scientific, Bremen, Germany)). The eluents of each sample were collected and lyophilized. Then, 100 µL of 0.1 M TEAB buffer was added to reconstitute the samples, and 41 µL of acetonitrile-dissolved TMT labeling reagent was added. The samples were mixed with shaking for 2 h at room temperature, after which the reaction was stopped by adding an equal volume of 8% ammonia. All labeled samples were then desalted and lyophilized.

#### 2.4.3. Fraction Separation

Gradient elution was performed using mobile phases A (2% acetonitrile, pH adjusted to 10.0 using ammonium hydroxide) and B (98% acetonitrile). The lyophilized samples were first dissolved in mobile phase A and then centrifuged at 12,000× *g* for 10 min at room temperature. The samples were fractionated in a Rigol L3000 HPLC system (Rigol, Beijing, China) using a C18 column (Waters BEH C18, 4.6250 mm, 5 m). The column oven was 45 °C, and the flow rate was 1 mL/min. The specific elution gradient was as follows: 0–10 min, 97–95% mobile phase A; 10–30 min, 95–80% mobile phase A; 30–48 min, 80–60% mobile phase A; 48–50 min, 60–50% mobile phase A; 50–53 min, 50–30% mobile phase A; and 53–54 min, 30–0% mobile phase A. The eluates were observed at UV 214 nm, collected at one tube per minute, and then combined to form 10 fractions. All fractions were vacuum-dried and then reconstituted in 0.1% (*v*/*v*) formic acid.

#### 2.4.4. Liquid Chromatography Tandem Mass Spectrometry (LC-MS/MS) Analysis

Shotgun proteomics analyses for the creation of transcription libraries was carried out using a Q ExactiveTM HF-X mass spectrometer (Thermo, Bremen, Germany) in data-dependent acquisition mode and an EASY-nLC^TM^ 1200 UHPLC system (Thermo Fisher, Bremen, Germany). Each sample (1 µg) was injected into a Dr. Maisch GmbH C18 Nano-Trap column (analytical column packing 1.9 µm, precolumn packing 3 µm, pore size 120 A). The following liquid chromatography elution conditions were used to separate the peptides: 0–2 min, 94–85% mobile phase A; 2–78.5 min, 85–60% mobile phase A; 78.5–80.5 min, 60–50% mobile phase A; 80–81.5 min, 50–45% mobile phase A; and 81.5–90 min, 45–0% mobile phase A. The flow rate was 600 nL/min. The peptides were examined using the Q Exactive HF-X mass spectrometer with a Nanospray Flex^TM^ ion source (ESI) (Thermo Fisher, Bremen, Germany), a spray voltage of 2.3 kV, and an ion-transport capillary temperature of 320 °C. Full scanning was conducted from *m*/*z* 350 to 1500 with a resolution of 60,000 (at *m*/*z* 200), an automatic gain control (AGC) target value of 3 × 10^6^, and a maximum ion injection time of 20 ms. The top 40 most abundant precursors in the full scan were fragmented by higher-energy collisional dissociation and analyzed by MS/MS with a 10 plex resolution of 45,000 (at *m*/*z* 200) and an AGC target value of 5 × 10^4^. The maximum ion injection time was 86 ms, the normalized collision energy was 32%, the intensity threshold was 1.2 × 10^5^, and the dynamic exclusion parameter was 20 s. Mass spectrum detection raw data were generated. 

#### 2.4.5. Metabolite Profiling

The raw data from the LC-MS/MS analysis were uploaded into Proteome Discoverer 2.2 (PD2.2, Thermo, Bremen, Germany) for library search analysis. A precursor ion mass tolerance of 10 ppm and a product ion mass tolerance of 0.02 Da were used as search criteria. Carbamidomethyl was specified as a fixed modification. Methionine oxidation and TMT plex were specified as dynamic modifications. Acetylation and TMT plex were listed as N-terminal modifications. A maximum of two miscleavage sites was permitted. To improve the quality of the analysis results, PD2.2 software was used to further filter the search results as follows: peptide–spectrum matches (PSMs) with a confidence level of 99% or higher were considered plausible PSMs, proteins containing at least one unique peptide were considered plausible proteins, only plausible peptides and proteins were retained, and FDR verification was conducted to remove peptides and proteins with an FDR greater than 1%.

The protein quantitation results were statistically analyzed using a *t*-test. Proteins that were significantly different between the experimental and control groups (*p* < 0.05, fold change (FC) > 1.5 or FC < 0.67) were defined as differentially expressed proteins (DEPs). Interproscan (5.18–57.0 (http://www.ebi.ac.uk/interpro/interproscan.html, accessed on 19 February 2023)) software was used to conduct Gene Ontology (GO). The DEPs were analyzed for functional protein families and pathways using Cluster of Orthologous Groups of proteins (COG) and the Kyoto Encyclopedia of Genes and Genomes (KEGG). Pathway enrichment analysis was performed using GO and KEGG, and the DEPs were analyzed using cluster heat maps and volcano maps.

## 3. Results

### 3.1. Effect of Amantadine on the Histomorphology of the A. japonicus Intestinal Tract

**Figure 1:**
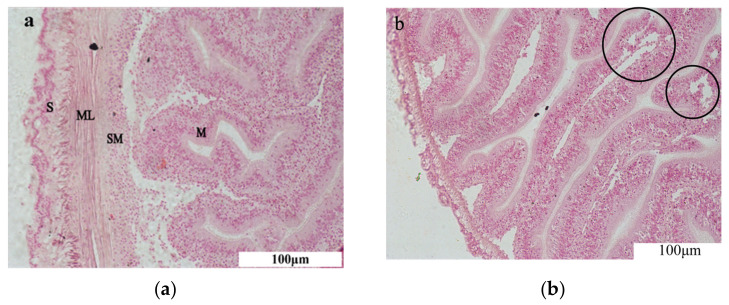
Histological observation of the intestine. (**a**) Control group. (**b**) Experimental group. S: plasma membrane layer; ML: muscle layer; SM: inner connective tissue layer, i.e., submucosa; M: intestinal luminal epithelium, i.e., mucosa. Black circles indicate epithelial cell necrosis. Scale bar = 100 μm.

The four layers, from the inside out, that comprise the digestive tissue of *A. japonicus* are the mucosal layer, submucosal layer, muscular layer, and plasma membrane layer (Figure 1). The mucosal layer is composed of mucous cells and columnar or cuboidal cells, and it can be a single layer or pseudostratified. Loose connective tissue comprises the submucosal layer. The muscular layer is divided into two layers—the inner longitudinal layer and outer ring—and the plasma membrane layer consists of flattened cells and a thin layer of connective tissue beneath them. After amantadine exposure, the intestinal plasma membrane layer was thinned and partially disintegrated. The muscular layer was thinned, the submucosal connective tissue was thinner and had partially disappeared, and the mesothelial cells were slightly swollen. Folds were still present, but the gaps between the folds were enlarged. Furthermore, the epithelial cells showed massive necrosis.

### 3.2. Effect of Amantadine on Intestine Oxidative Stress Response

**Figure 2:**
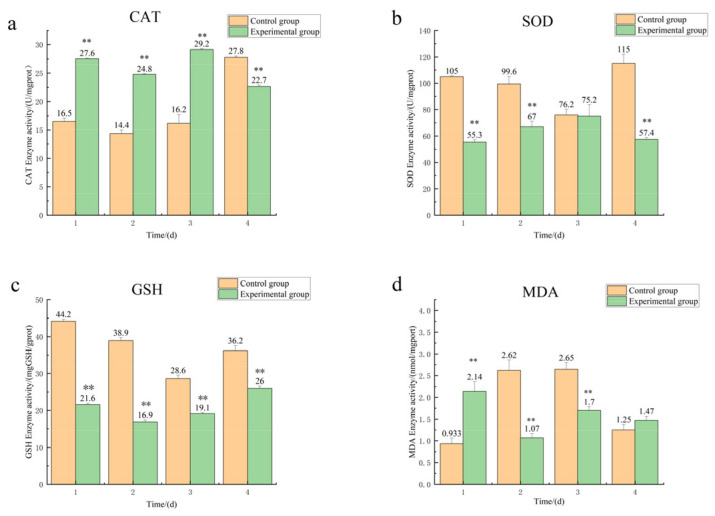
Enzyme activities of (**a**) catalase (CAT), (**b**) superoxide dismutase (SOD), (**c**) glutathione (GSH), and (**d**) malondialdehyde (MDA) in the intestinal tract. Note: ** means that there is a significant difference compared with the control group (*p* < 0.01).

The intestinal CAT enzyme activities after amantadine exposure are shown in Figure 2a. From days 1 to 3, the CAT enzyme activities of the experimental group were significantly (*p* < 0.01) higher than those of the control group and reached their highest levels on day 3, indicating significant induction. Figure 2b shows the intestinal SOD enzyme activities after amantadine exposure. The SOD enzyme activities in the experimental group were always lower than those of the control group at each time point, but showed an overall increasing and then a decreasing trend. The SOD enzyme activities of the experimental group gradually increased from days 1 to 3, and on day 3 were similar to those of the control group; on day 4, the SOD enzyme activities in the experimental group decreased and were significantly lower than those of the control group (*p* < 0.01). Figure 2c shows that the GSH contents in the intestines of the experimental group were always significantly lower than those of the control group (*p* < 0.01). However, there was an overall trend of decreasing and then increasing. The intestinal GSH contents in the experimental group gradually decreased from days 1 to 2, but from days 2 to 4 gradually increased. The MDA contents of the intestines after amantadine exposure are shown in Figure 2d. On days 1 and 4, the MDA contents in the experimental group were higher than those of the control group, indicating MDA induction. However, on days 2 and 3, the MDA contents in the experimental group were significantly lower than those of the control group.

### 3.3. Proteomic Response in the A. japonicus Intestinal Tract after Amantadine Exposure

#### 3.3.1. Protein Data Reliability

After the mass spectrometry data retrieval, we evaluated the search results in order to examine the quality of the data [18,19,20,21]. Protein was extracted from the intestinal tissues of *A. japonicus*, and the protein concentrations and total protein contents were determined using a kit (Table 1).

**Figure 3:**
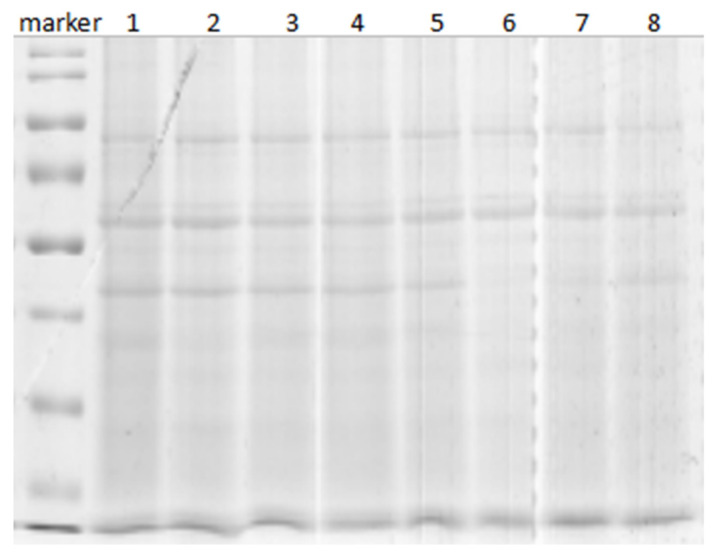
SDS-PAGE electrophoresis of protein samples. Note: Lanes 1–4 were control group samples; lanes 5–8 were experimental group samples.

The SDS-PAGE results for eight samples are shown in Figure 3. The molecular weights of the total protein samples were distributed from large to small, and their molecular weights were generally greater or close to 50 kDa. The clarity of the bands indicated non-degraded protein. The bands for each group were similar, indicating that the differences in the electrophoresis results among the samples were not significant. Therefore, the protein samples were of sufficient quality for use in subsequent experiments.

In order to improve the quality of the analysis results and reduce the false-positive rate, further filtering of the retrieval results was performed using Proteome Discoverer software: peptide–spectrum matches (PSMs) with a confidence of 99% or higher were considered to be trustworthy PSMs; proteins containing at least one unique peptide segment were considered to be trustworthy proteins; only reliable peptides and proteins were retained, and FDR validation was performed to remove peptides and proteins with an FDR greater than 1%.

**Figure 4:**
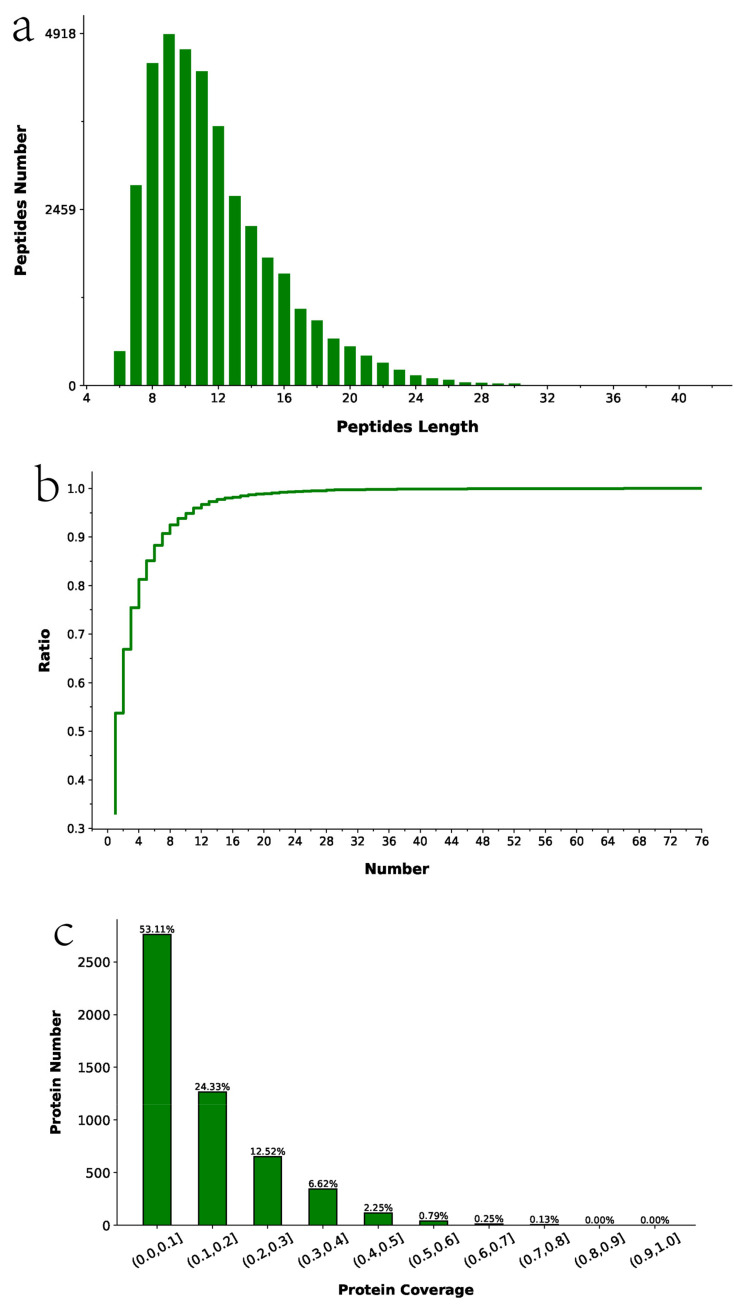

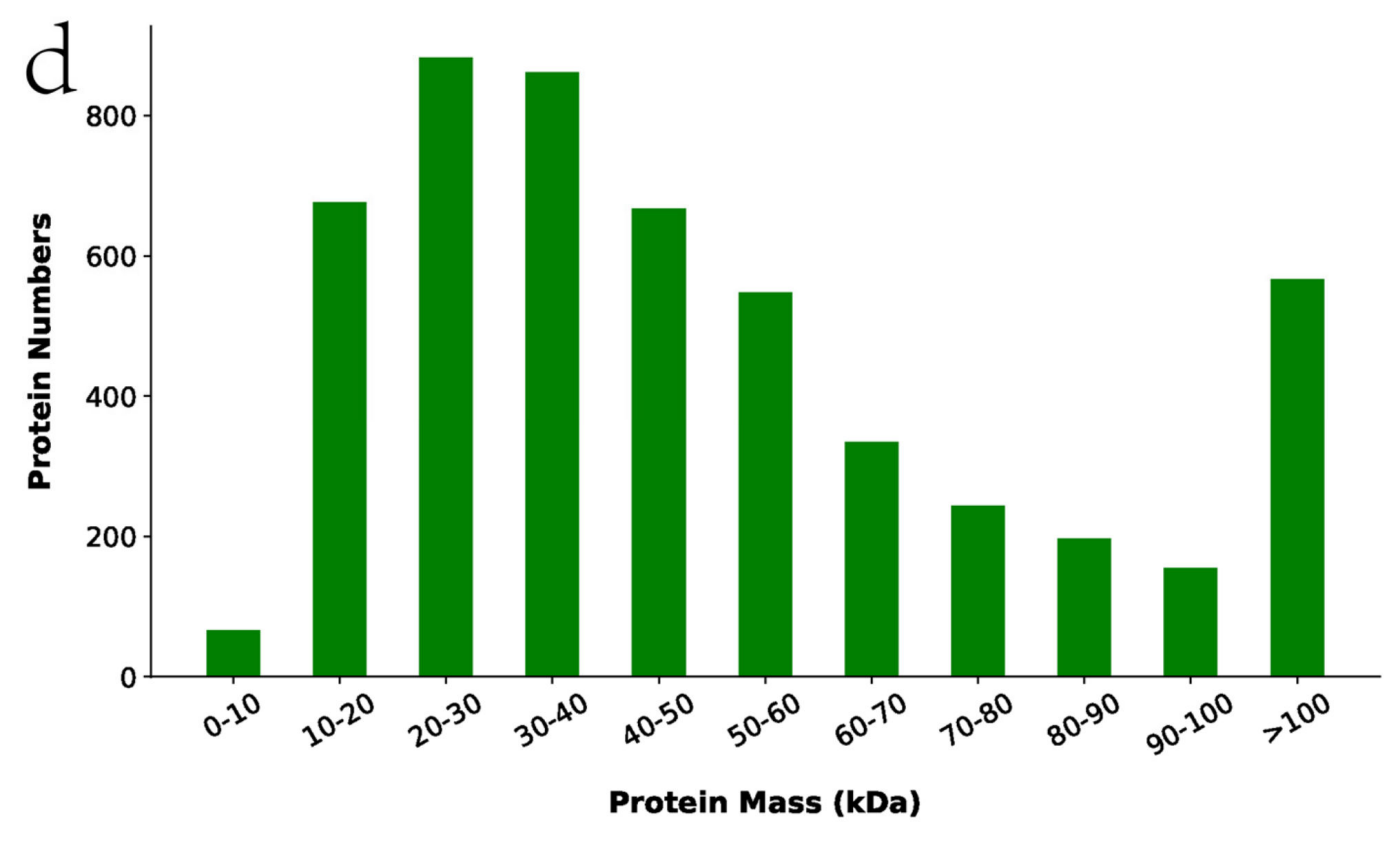
Proteomics data quality control. (**a**) Peptide length distribution of identified peptides. (**b**) Distribution of the number of unique peptide segments. (**c**) Protein coverage distribution. (**d**) Protein molecular weight distribution.

A total of 5190 proteins were identified by TMT labeling, mass spectrometry detection, and annotation from the database. Data quality control was performed on the proteomics data. The results in Figure 4a show that most of the identified peptides contained 7–20 amino acids. The peptides were within a reasonable range of peptide lengths and were generally not too short or too long. Figure 4b shows a higher number of unique peptide sequences, indicating reliable identification of the proteins. Figure 4c shows that there was a wide protein coverage, and Figure 4d shows that there was a wide range of molecular weights. The identified proteins were shown to be reliable based on the peptide length distribution, the number of unique peptides, and protein coverage.

#### 3.3.2. Differential Protein Screen

**Figure 5:**
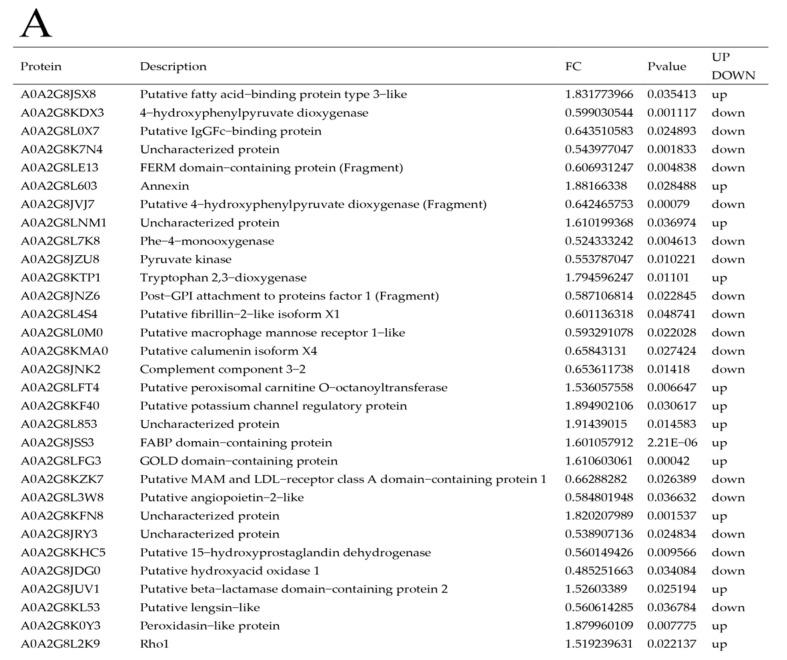

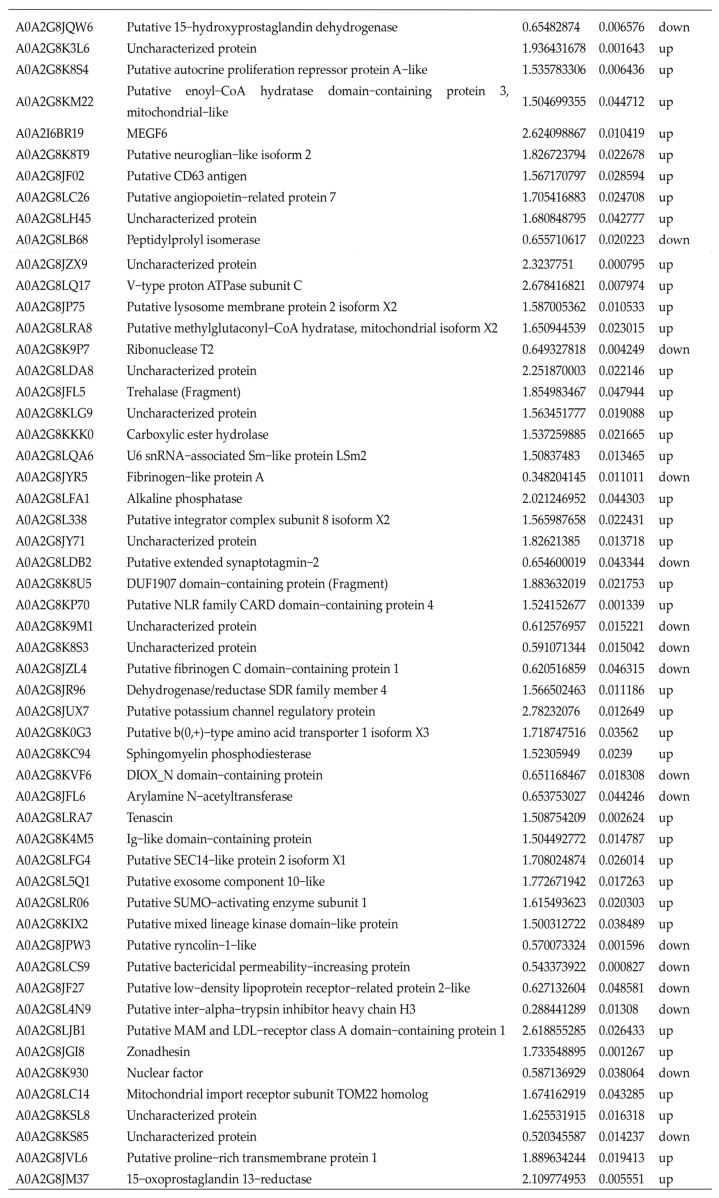

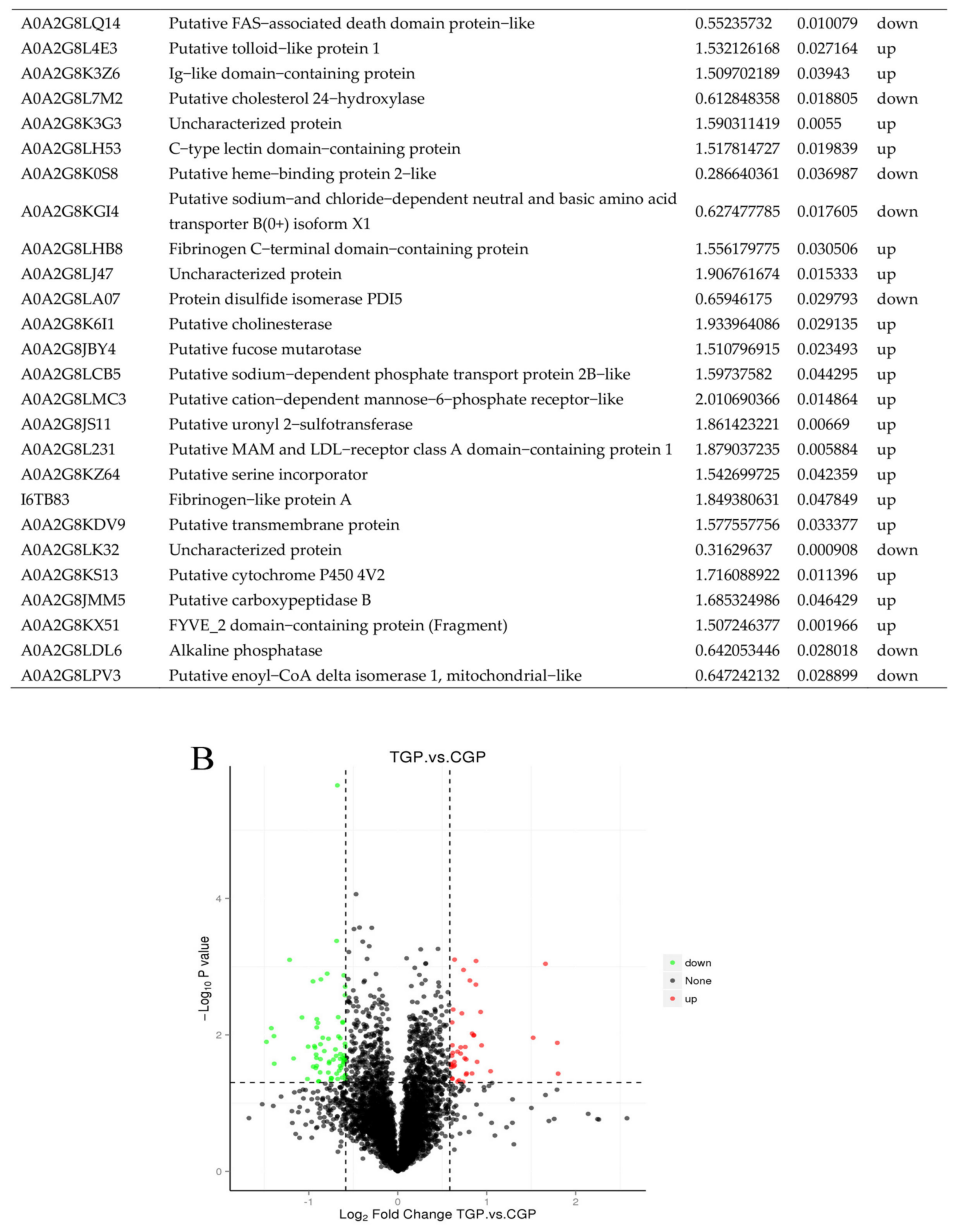
DEPs in *A. japonicus* after exposure to amantadine (**A**) and a differential protein volcano map (**B**).

The 5190 identified proteins were screened for upregulated expression (FC ≥ 1.5, *p* ≤ 0.05) and downregulated expression (FC ≤ 0.67, *p* ≤ 0.05). The results are shown in Figure 5. Out of a total of 111 DEPs, 69 were downregulated (represented by green dots) and 42 were upregulated (represented by red dots).

#### 3.3.3. Biological Pathway Analysis of DEPs

**Figure 6:**
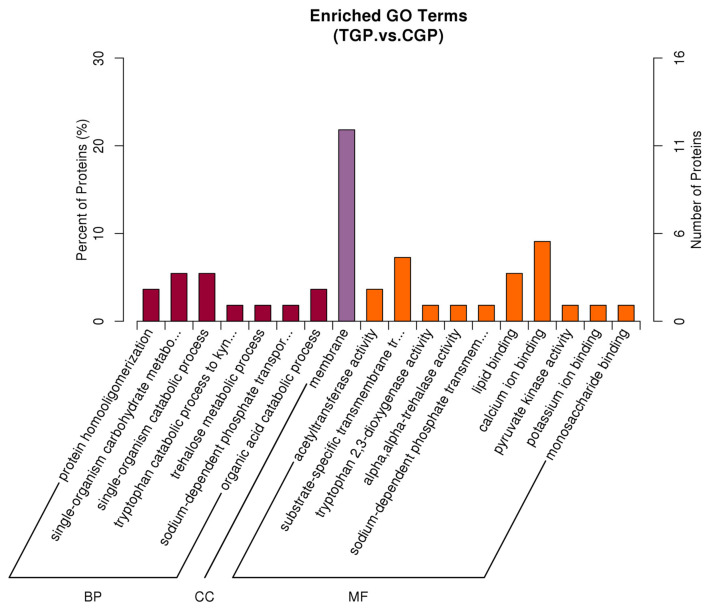
Gene Ontology enrichment analysis of differentially expressed proteins.

GO analysis of DEPs enables the identification of their main biological functions. As shown in Figure 6, the DEPs were significantly enriched in biological processes, cellular components, and molecular functions. Among the biological processes, the DEPs were mainly associated with protein homologation, single biological processes (metabolic processes), and organic acid catabolism. Among the cellular components, the DEPs were associated with cell membranes. Among the molecular functions, the DEPs were involved in acetyltransferase activity, lipid binding, and calcium ion binding.

**Figure 7:**
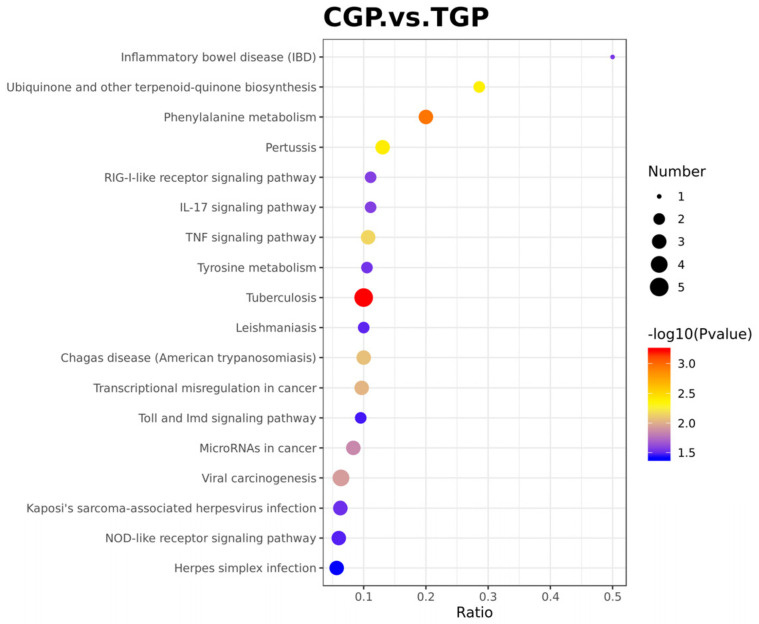
Kyoto Encyclopedia of Genes and Genomes pathways of the differentially expressed proteins.

GO enrichment analysis was used to identify the most significant biochemical metabolic pathways and signal transduction pathways in which the DEPs were involved. The top 20 enriched pathways are shown in Figure 7. Phenylalanine metabolism, tyrosine metabolism, inflammatory bowel disease, and Toll and Imd signaling were significantly enriched.

## 4. Discussion

Biomarkers of biological antioxidant enzyme system status were evaluated (SOD and CAT activities and MDA contents); these markers are widely used in environmental pollution research and ecotoxicological studies. SOD activity was lower in the experimental group than in the control group from day 1 to day 4, indicating that SOD activity was significantly inhibited by amantadine exposure. Excessive superoxide anions (O_2_^−^) can be assumed to be produced by *A. japonicus* in response to high concentrations of amantadine. The tissues showed signs of oxidative stress damage because the superoxide could not be cleared quickly enough by antioxidant enzymes, such as SOD, indicating that SOD activity was inhibited. CAT activity was significantly higher in the experimental group than in the control group in the first 3 days of exposure. This increased CAT activity might have been because of the superfluous amounts of superoxide anions converted to H_2_O_2_ in *A. japonicus*. Acute detoxification through enzymatic activity is probably an adaptive response to amantadine exposure. However, with prolonged exposure time, *A. japonicus* might have experienced immune fatigue or toxicity, resulting in significantly lower CAT activity in the experimental group compared with the control group on day 4. These results are similar to those of Tian et al. [22].

The most important antioxidant substance in marine organisms is GSH because it is used to control redox reactions. Organisms reduce oxidative damage by decreasing the GSH content in cells [23,24]. We found that GSH contents were significantly lower in the experimental group than in the control group from days 1 to 4. Increasing the GSH activity in response to amantadine exposure would help to eliminate reactive oxygen species (ROS), thus reducing oxidative damage in the organism. Studies have found that MDA contents in organisms are elevated when they are exposed to contaminants [25,26]. Here, MDA contents increased rapidly in the experimental group and peaked on the first day of exposure and then decreased on days 2–4. The rapid increase in MDA activity was likely owing to the oxidative stress caused by amantadine. The organism would subsequently eliminate MDA by activating other antioxidant enzymes to maintain the stability of the antioxidant system.

Carbohydrates are the main components of cell structures, the main source of energy, and play an important role in regulating cell activity [27]. The results showed that pyruvate kinase was increased through glycolysis, gluconeogenesis, and pyruvate metabolism after amantadine exposure. Pyruvate kinase converts phosphoenolpyruvate (PEP) to pyruvic acid in the pyruvate metabolic pathway. Glucose homeostasis also plays an important role in the energy needed for growth and development. In glycolysis, glucose is converted to pyruvate through a series of catalytic reactions that produce small amounts of ATP in the cytoplasm. The key enzyme in this process is pyruvate kinase, which regulates the last step of glycolysis by catalyzing the reaction between PEP and ADP to produce pyruvate and ATP [28]. After amantadine exposure, *A. japonicus* increased energy production and conversion by regulating the glycolytic and glycogenic pathways.

Proteins and amino acids are key molecules that play important roles in the structure and metabolism of all organisms. We found that the valine, leucine, and isoleucine degradation pathways were altered. Valine, isoleucine, and leucine are essential branched-chain amino acids (BCAAs). High concentrations of BCAAs can stimulate muscle protein synthesis and promote muscle growth. Several studies have shown that adding leucine [29,30,31], isoleucine, and valine to the diet can improve muscle growth in organisms. Therefore, the degradation of valine, leucine, and isoleucine after amantadine exposure may have indicated that *A. japonicus* growth was inhibited. The essential amino acid phenylalanine also has a marked effect on protein synthesis and physiological metabolism in aquatic animals. Guo et al. [32] discovered that appropriate levels of phenylalanine could maximize protein synthesis and improve the growth of organisms. Phenylalanine can be transformed into tyrosine by phenylalanine hydroxylase in the phenylalanine metabolic pathway. We found that phenylalanine hydroxylase levels were increased after amantadine exposure, suggesting that the conversion of phenylalanine to tyrosine was increased and that protein synthesis was reduced owing to decreased phenylalanine concentrations, which would have inhibited *A. japonicus* growth.

NF-κB is a protein complex that regulates DNA transcription. NF-κB is an inducible transcription factor that regulates many genes involved in inflammation (including adhesion molecules), the immune response, cell proliferation, and apoptosis [33]. The results showed that the NF-κB, tumor necrosis factor (TNF), and IL-17 (interleukin-17) signaling pathways were affected by amantadine exposure. NF-κB is redox-sensitive and can transactivate several genes involved in the inflammatory response; this leads to the activation of multiple inflammation-related metabolic pathways that can further activate NF-κB in response to oxidative stress and increase the production of proinflammatory cytokines [34]. The NF-κB signaling pathway is considered to be a central pathway in inflammatory bowel disease; this pathway is induced by multiple signals and plays a key role in the subsequent expression of proinflammatory cytokines [35]. TNF is commonly referred to as TNFα; it is a cytokine that is pleiotropic for various cell types and triggers a range of inflammatory molecules, including other cytokines and chemokines. TNF has been identified as the main regulator of the inflammatory response and is involved in some inflammatory and autoimmune diseases. When TNF binds to TRADD, TRADD recruits other mediating proteins, including receptor-interacting protein 1 (RIP1) and TNFα receptor-associated factor 2 (TRAF2), which form a complex that activates NF-κB [36]. IL-17 is a proinflammatory cytokine that activates NF-κB by mediating Act 1 and TRAF 6. Il-17 induces the transcription of proinflammatory protein genes, including cytokines, chemokines, and matrix metalloproteinases, to amplify inflammation [37]. Priya et al. [38] showed that NF-κB was activated by signaling pathways and by ROS to trigger the inflammatory response in the body. Our results suggest that amantadine activated NF-κB through multiple signaling pathways and through ROS. The activated NF-κB then promoted the production of inflammatory factors, which in turn amplified inflammation through the IL-17 pathway, resulting in intestinal inflammation. In addition to the production of proinflammatory factors, NF-κB activation can induce apoptosis. Cell apoptosis is induced by TNFα first binding to TRADD, which recruits FADD and procaspase-8 to form a complex as part of the TNF signaling pathway [34]. The NF-κB pathway mediates heterodimerization of the p50 and p65 subunits of the NF-κB family linked to the repressor protein Iκ-B (a regulator of anti-apoptotic genes). Thus, NF-κB p65 is essential for maintaining the balance between cell death and survival. We found increased levels of NF-κB p50, indicating the increased synthesis of heterodimers of the p50 and p65 subunits, which would promote apoptosis in the intestinal tract of *A. japonicus*. Therefore, amantadine may activate NF-κB by inducing multiple signaling pathways. Activated NF-κB could then promote the production of inflammatory factors, causing intestinal inflammation and apoptosis in *A. japonicus*. Amantadine probably activated NF-κB via the TNF and NF-κB signaling pathways, and the activated NF-κB led to intestinal cell apoptosis.

## 5. Conclusions

This study showed that energy production and conversion can be increased through the glycolysis and gluconeogenesis pathways. Growth was regulated through the valine, leucine, isoleucine, and phenylalanine metabolic pathways. In addition, after amantadine exposure, ROS were produced in the intestinal tissues of *A. japonicus*. Oxidative stress caused by excess ROS production was mitigated by activating SOD, GSH, and CAT enzymes. Meanwhile, intestinal NF-κB was likely activated by excess ROS and the NF-κB signaling pathway, and the activated NF-κB triggered inflammation in the *A. japonicus* intestine. The inflammation was amplified by the IL-17 signaling pathway. Observation of the tissue sections showed that the intestinal plasma membrane layers and connective tissues were thinned and partially disintegrated after exposure to high concentrations of amantadine. The muscle layers were thinned, the mesothelial cells were slightly swollen, the fold gaps were enlarged, and the epithelial cells showed massive necrosis. The findings indicate that apoptosis was induced by the activation of NF-κB in *A. japonicus* intestinal cells via the NF-κB and TNF signaling pathways (Table S1).

## Figures and Tables

**Table 1:** Protein Concentrations and Total Protein Contents.

Serial Number	Sample Name	Protein Concentration (μg/μL)	Total Protein
1	CGP-1	3.84	7660.80
2	CGP-2	3.82	7620.90
3	CGP-3	4.38	8738.10
4	CGP-4	4.05	12,129.75
5	TGP-1	4.10	8179.50
6	TGP-2	3.13	6228.70
7	TGP-3	3.00	5970.00
8	TGP-4	4.25	8478.75

Note: CGP1-4 was the control group; TGP1-4 was the experimental group.

## Data Availability

All data are presented in the text. They are also available on request from the corresponding authors.

## Supplementary Materials

The following supporting information can be downloaded at: https://www.mdpi.com/article/10.3390/toxics11030226/s1, Table S1: Changes in dipeptide metabolites in *A. japonicus* after amantadine exposure.
