# Supplementary material for: Amantadine Toxicity in Apostichopus japonicus Revealed by Proteomics
Source: Toxics. 2023 Feb 27;11(3):226. doi: 10.3390/toxics11030226 (PMC10053536; doi:10.3390/toxics11030226)
Supplement: Supplementary file 1 [file toxics-11-00226-s001.zip › toxics-2120618-supplementary.pdf]

toxics

Amantadine Toxicity in *Apostichopus japonicus* Revealed by Proteomics

Junqiang Zhao [1,2,†], Jianqiang Chen [1,†], Xiuhui Tian [1], Lisheng Jiang [1], Qingkui Cui [1], Yanqing Sun [1], Ningning Wu [3], Ge Liu [4], Yuzhu Ding [1], Jing Wang [1], Yongchun Liu [1], Dianfeng Han [1,*] and Yingjiang Xu [1,*]

Table S1. Changes in dipeptide metabolites in *A. japonicus* after amantadine exposure.

Name	Formula	FC	log2FC	*p*-Value	ROC	VIP	Up_ Down
Val-Ser	$C_8H_{16}N_2O_4$	2.918265786	1.545111285	0.001042874	1	1.769000258	up
N-glycyl-L-proline	$C_5H_{10}N_2O_4$	3.136754646	1.649272688	0.004054509	1	1.658107507	up
Glycyl-L-leucine	$C_8H_{16}N_2O_3$	4.005500023	2.001982351	0.02060531	1	1.555792644	up
